# Neural Schematics as a unified formal graphical representation of large-scale Neural Network Structures

**DOI:** 10.3389/fninf.2013.00022

**Published:** 2013-10-24

**Authors:** Matthias Ehrlich, René Schüffny

**Affiliations:** Highly-Parallel VLSI-Systems and Neuromorphic Circuits, Institute of Circuits and Systems, Technische Universität DresdenDresden, Germany

**Keywords:** Neural network structures, cortical microcircuits, neural schematics, neural modeling, neural simulation, PyNN

## Abstract

One of the major outcomes of neuroscientific research are models of Neural Network Structures (NNSs). Descriptions of these models usually consist of a non-standardized mixture of text, figures, and other means of visual information communication in print media. However, as neuroscience is an interdisciplinary domain by nature, a standardized way of consistently representing models of NNSs is required. While generic descriptions of such models in textual form have recently been developed, a formalized way of *schematically* expressing them does not exist to date. Hence, in this paper we present *Neural Schematics* as a concept inspired by similar approaches from other disciplines for a generic two dimensional representation of said structures. After introducing NNSs in general, a set of current visualizations of models of NNSs is reviewed and analyzed for what information they convey and how their elements are rendered. This analysis then allows for the definition of general items and symbols to consistently represent these models as *Neural Schematics* on a two dimensional plane. We will illustrate the possibilities an agreed upon standard can yield on sampled diagrams transformed into *Neural Schematics* and an example application for the design and modeling of large-scale NNSs.

## Introduction

One of the major outcomes of neuroscientific research are models of *Neural Network Structures* (NNSs) that have to be communicated to the research community. In scientific publications, descriptions of these models usually consist of a non-standardized mixture of text, figures, and other means of visual information communication in print media (Nordlie et al., 2009). However, as neuroscience is an interdisciplinary domain by nature where researchers from biology, psychology, mathematics, physics, computer science, or electrical engineering have to cooperate, a standardized way of consistently representing neural network models is required to communicate their concepts without obstacles. But whereas generic descriptions of such models in textual form have recently been developed (Davison et al., 2009; Gleeson et al., 2010), graphical representations of neural structures in the research field of neuroscience remain diverse.

Nordlie et al. noted that *“one may choose from a [] variety of styles for [] diagrams, and it is not a priori clear which style is best.”* (cf. Nordlie et al., 2009, p. 15) and stated that (computational) neuroscientists *“mostly [rely] on box-and-arrow diagrams [] using ad hoc notations with conflicting use of symbols”* (cf. Nordlie and Plesser, 2010, p. 1). Consequently they presented *Connectivity Pattern Tables* (CPTs) as a means to generalize representations of the connectivity of neural networks. In the same line of argument, Djurfeldt (2012) presented the *Connection-set Algebra* (CSA) as another approach to express neural network connectivity without ambiguity.

We herewith propose *Neural Schematics* as a generic two dimensional schematic representation of large-scale NNSs. The idea of *Neural Schematics* is inspired by similar approaches from engineering as well as *de-facto* standards from natural sciences. An example from schematics in engineering are standards in electrical engineering such as the IEEE^1^ Std 315-1975 *“Graphic Symbols for Electrical and Electronics Diagrams”* (IEEE, 1975) or the IEEE Std 91-1984 *“Graphic Symbols for Logic Functions”* (IEEE, 1984) and its application in the graphical representation of electric and electronic circuitry. These schematics are made editable via schematic editors and have corresponding textual representations such as the *Hardware Description Languages* (HDLs) like SystemC (IEEE, 2005), VHDL (IEEE, 1999) or SystemVerilog (IEEE, 2012). For the natural sciences we might look at systems biology with the *Systems Biology Graphical Notation* (SBGN) (Novere et al., 2009), its textual form *Systems Biology Markup Language* (SBML)(Hucka et al., 2003), and editors that enable schematic editing such as, for example, the CellDesigner (Funahashi et al., 2008).

To develop the concept of *Neural Schematics* we will first describe in *Materials and Methods* a general modeling approach for *Neural Network Structures* NNSs to introduce a common terminology. This is followed by a summary of the selection process of diagram samples depicting *Neural Network Structures* NNSs and the specification of the elements we will scan NNS samples for. A set of guidelines for the development of the *Neural Schematics* concludes this section.

As *Results* we will first search the sampled figures as well as their references for the structural information provided and then compare these findings to determine what the *Neural Schematics* should comprise. These results are subsequently used to develop the concept of the *Neural Schematics*. As proof-of-concept a subset of the samples is then redrawn and a mock-up of a modeling software tool is presented to illustrate a further application for the *Neural Schematic* concept. As a textual representation of the *Neural Schematics* is essential for the concept's diffusion into the research community, we furthermore reason and suggest the use of PyNN as such. In the last section we will conclude with a discussion of the concept and its applications.

## Materials and methods

To guide the analysis of schematic drawings of NNSs, we establish a common terminology by describing a modeling approach for NNSs (1). Afterwards the sample selection and analysis process is specified (2) and definition guidelines for the symbols and elements of *Neural Schematics* are stated (3).

### Modeling large-scale neural network structures

A model of a NNS describes in an abstract manner a functional principle of the *Central Nervous System* of the vertebrate brain. We assume a *Cortex* centered modeling approach and thus other regions of the brain are *Non-Cortical Regions* (NCRs).

From a cytological point of view the Cortex is a structure made up of *layers* of tissue with a layering perpendicular to its surface. These layers are differentiated by the morphological distinctiveness and connectivity of the neurons they contain (Ramón y Cajal, 1904) and are numbered starting below the Cortex's surface with Layer I counting upward when moving down into the Cortex's matter to Layer VI as the lowest.

The surface of the Cortex can be partitioned into *areas* as first introduced by Brodmann (1909). This division, which was primarily based on histological criteria, was later proven as also functionally related and is still being refined today (Wallace et al., 2004). Dedicated areas handle a specific information processing task. Among the areas there is a hierarchy according to the complexity of the task, starting from lower cortical areas doing preprocessing to the higher cortical areas performing associative tasks.

Elements in the Cortex or NCRs might be grouped into functional *units* that can subdivide an area or NCR, such as the Cortical Column (Mountcastle, 1997) that subdivides areas in the Cortex. A unit is a canonical hierarchical element, i.e., it can be a super-unit or sub-unit to other units.

A compound of functionally related neurons that belong to the same *morphological/electrophysiological* cell class might in a model be grouped together to a *population*, as for example in Douglas et al. (1998) or Haeusler and Maass (2007). These populations are connected via *projections*, with a projection leaving a population as *efferent* and entering a population as *afferent*. A projection bundles synaptic connections between neurons of a population. All synaptic connections of a projection are of the same excitation type,^2^ which are either *excitatory* or *inhibitory*.

Populations and projections might have additional attributes. Additional attributes related to the group of neurons are for instance neuron model parameters and spatial distribution of the neurons. For a projection, additional attributes might be the weights, dynamics, and delays of its synaptic connections as well as its synaptic connection density.

### Sample selection and analysis process

We will analyze a set of sample diagrams to determine in detail what these have in common and, hence, what should be expressible with standardized schematic elements.

The samples resemble a merely randomly drawn set of six neural network models that are scanned for the information they convey and what graphical elements the authors utilized for that purpose. For a comparative analysis we will primarily but not exclusively search for elements as introduced with the description of the modeling approach, such as:

- populations,
- neuronal attributes,
- projections,
- synaptic attributes,
- layers,
- areas,
- NCRs,
- units.

Secondly, we will look for any additional attributes of projections and populations or any other structural information provided in a figure or in its reference. By comparison of the found features we can then isolate the set of elements required for *Neural Schematics*.

### Definition guidelines

For the evaluation of the rendering styles of the sample diagrams and the definition of symbols and elements for *Neural Schematics* we have chosen the following guidelines: (1) base the concept upon the schematic style for electrical circuitry, (2) favor simplicity over visual distinctiveness, and (3) with application primarily for visual modeling in CAD^3^ applications and secondly for figures in publications.

## Results

The concept of *Neural Schematics* is now developed stepwise. First the sampled graphical NNS representations are introduced and analyzed (1). Subsequently we compare the analysis results to isolate the required *Neural Schematic* features (2). Following the comparison we define items and symbols to graphically represent said features on a two dimensional plane (3) and then redraw a subset of the sampled NNSs (4). An illustrative application for *Neural Schematics* and a proposal for its textual representation (5) conclude the results.

### Sample analysis

**Figure 1:**
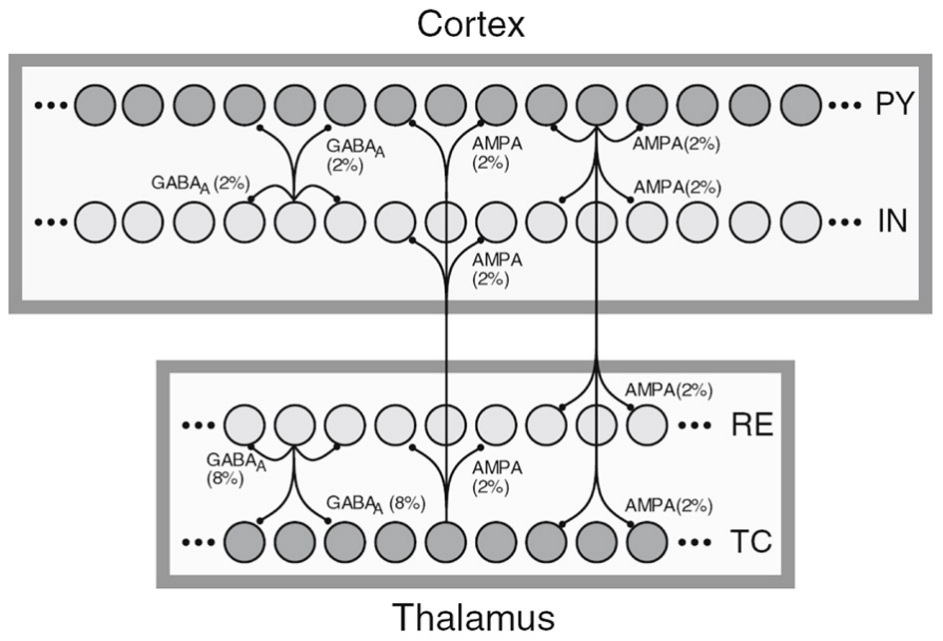
**A schematic of Thalamocortical regions, taken from Destexhe (2009)**.

The first sample, shown in Figure 1, depicts a general NNS of *Thalamocortical* regions and was taken from Destexhe (2009). Squares represent different regions such as the *Cortex* or the NCR of the *Thalamus*. Circles within the regions stand for single neurons with inter- and intra-regional projections rendered as directed edges between single neurons. A row of circles represents a population of cells, with a label on the right side of the population holding its cell type. Here PY stands for *Pyramidal* cells, IN for inhibitory *Interneurons*, TC for *Thalamocortical*, and RE for *Thalamic Reticular* neurons. The filling color of the circles representing neuron signifies the excitation type of the efferents with dark gray for excitatory and light gray for inhibitory. The edges are labeled with the projection's connection density in percent and a synapse receptor type. The latter is implicitly denoting a projection's excitation type as the pointer tips of the edges are the same for both types. Furthermore, a certain degree of locality of the connections is implied by the connectivity drawing style.

In the referenced publication the reader finds more details on the cell classes of the modeled NNS. Although information on regions is present in the image only, the publication provides layer and area information for the cortical fraction of the NNS, here *Layer VI* and *Area 5* of cat, designating the experimental basis for the connectivity of that NNS. No functional grouping into units can be found.

**Figure 2:**
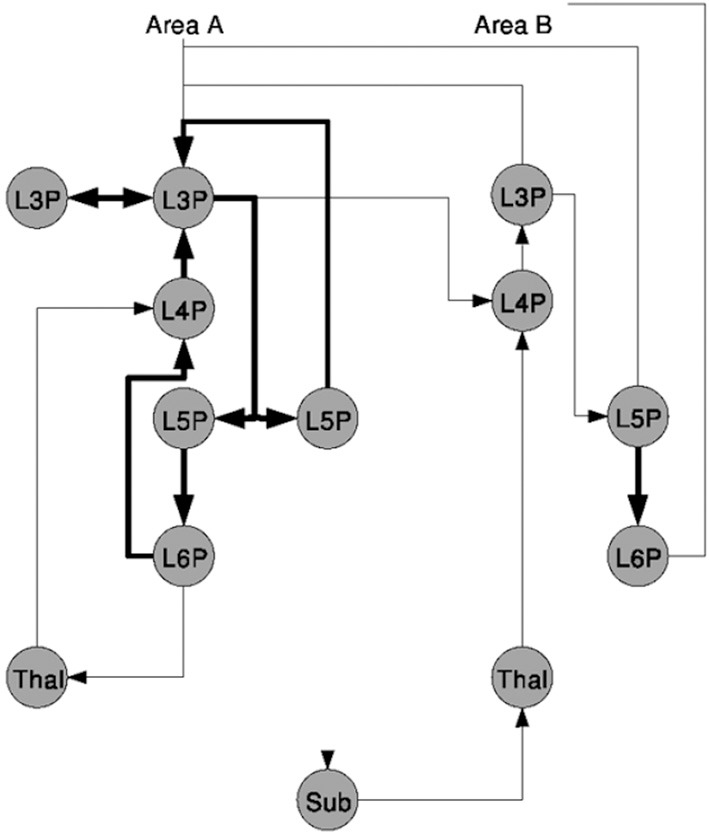
**A schematic of the microcircuitry of Thalamocortical regions, taken from Douglas and Martin (2004)**.

A second sample was taken from Douglas and Martin (2004) and is shown in Figure 2. The figure sketches a template for excitatory *Thalamocortical* microcircuitry primarily based on experimental data of *Area 17* or the *Primary Visual Cortex V1* of cat, see references in Douglas and Martin (2004). Circles represent different populations labeled with the layer Lx and the neuron type, e.g., P as pyramidal for the cortical populations and Thal as thalamic or Sub for other sub-cortical regions as NCRs. The vertical distribution of the nodes in the graph represents a population's area correspondence. Projections are exclusively excitatory and are drawn as directed arrows of which the ones rendered with thicker lines indicate local projections.

As a qualitative NNS such as the model communicated here requires less information to be present in the figure as for a more quantitative description, e.g., such as Figure 1, no additional information can be found in the image or in the reference.

**Figure 3:**
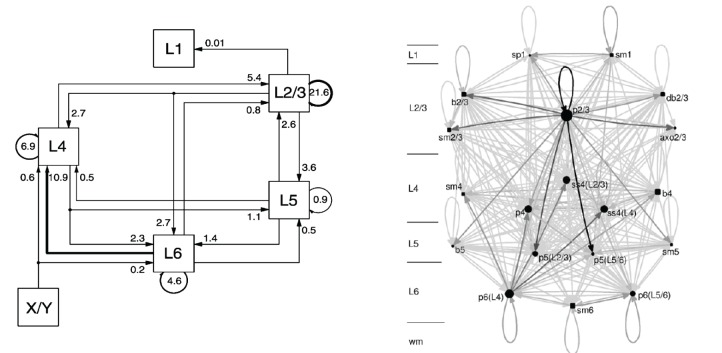
**A schematic of the Primary Visual Cortex V1 of cat on the left and its Cortical Graph on the right, taken from Binzegger et al. (2004) and from Binzegger et al. (2009), respectively**.

In Figure 3 two graphical representations of a NNS located in *Area 17* of the *Primary Visual Cortex V1* of cat including its X/Y afferents from the dorsal *Lateral Geniculate Nucleus* (LGN), as part of the NCR of the Thalamus, are shown that are both based on the same experimental data.

The schematic on the left was taken from Binzegger et al. (2004) and displays the fraction of the total synapses, that are involved in the excitatory projections between populations on different layers. The populations are rendered as boxes and in general labeled with the layer in which they are located. Arrows direct the projections which are all of the same excitatory type. A projection is visually attributed with its fraction of the total synaptic connections as a label. The projection with the largest fraction of synaptic connections is highlighted. In the reference such schematics are drawn likewise for projections between populations that are the source of inhibitory projections, for projections from populations that are the source of excitatory projections to populations that are the source of inhibitory projections and vice versa using the same projection style as for the image presented above, alternating the meaning of the arrow as either excitatory or inhibitory in text. The area for which the microcircuitry was modeled is only given in the reference. Morphological cell classes of the populations are also to be found in the reference where the receptor types and the connection densities are indicated.

Figure 3 on the right, taken from Binzegger et al. (2009), depicts a *Cortical Graph* of the same NNS. The graph shows the complete set of projections. An arrow representing a projection encodes in its gray-level the relative number of synapses between the populations. Populations that are the source of excitatory projections are pictured as square shapes and sources of inhibitory projections as circular shapes, with their sizes scaled according to the number of neurons per populations. The population labels denote the morphological cell class accompanied by the layer in which they are located, and for a subset the preferred layer(s) of axonal innervation is given in parentheses. An additional scale located left of the graph also indicates the layer of a population's spatial location.

**Figure 4:**
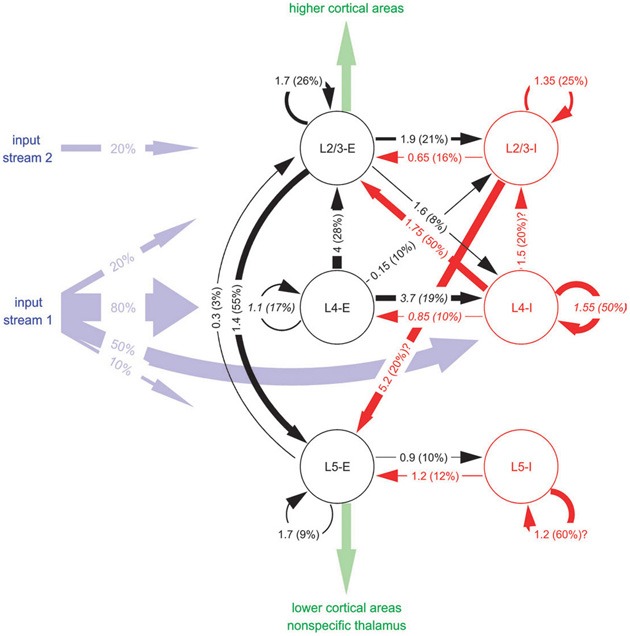
**A schematic of a Cortical Microcircuit Template, taken from Haeusler and Maass (2007)**.

The graph in Figure 4 depicts a *Cortical Microcircuit Template* by Haeussler and Maass sampled from Haeusler and Maass (2007). The graph is based on combined experimental data from different cortical areas of different vertebrate species. It shows the inhibitory and excitatory connectivity between populations on different layers. A projection arrow's thickness is determined by the mean amplitude of the *Postsynaptic Potential* (PSP) at the soma in [*mV*] as the first number and the connection probability between the populations in percent as the second number in parentheses. Populations are rendered as circular nodes. Node shapes are labeled starting with the layer in which they reside followed by the excitation type of its efferents. The reference gives further basic information on the morphological cell types by distinguishing between pyramidal and non-pyramidal cells. Different colors of the circles and arrows denote excitatory types (red) and inhibitory types (black) as well as afferent input “streams” (blue) and efferent intra-cortical or thalamic output (green). In addition, the maximum spatial intra-laminar distance between populations and information on the modeled synaptic plasticity is provided in the reference.

**Figure 5:**
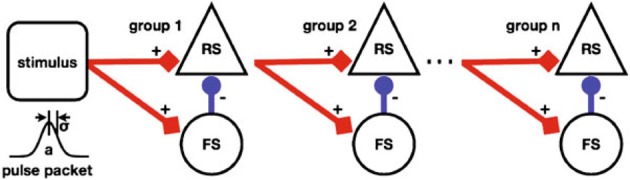
**A schematic of a Synfire Chain with Feed Forward Inhibition, taken from Kremkow et al. (2010)**.

Figure 5 depicts a *Synfire Chain* with *Feed Forward Inhibition* (FFI) of Kremkow et al. (2010) as a template of a NNS based on combined experimental data from different cortical areas of different vertebrate species.

The model depicts a horizontal chain where each chain element consists of a population of *Regular Spiking* (RS) neurons, rendered as triangle shaped nodes, that is inhibited vertically by a population of *Fast Spiking* (FS) neurons, the circular shaped nodes. The aggregation of populations to a chain element as a unit is labeled a group. The stimulus population of the chain is represented by a square shaped node visually accompanied by a diagram illustrating the stimulus generating function. Projections between populations are represented by arrows where the excitation types of the connections are differentiated by color, i.e., red for excitatory and blue for inhibitory, and shape of the pointer tip, i.e., square-cut for excitatory and circular for inhibitory, additionally labeled with + and −, respectively. Not shown in the figure but given in the reference are synaptic delays and connection densities as attributes of the projections.

**Figure 6:**
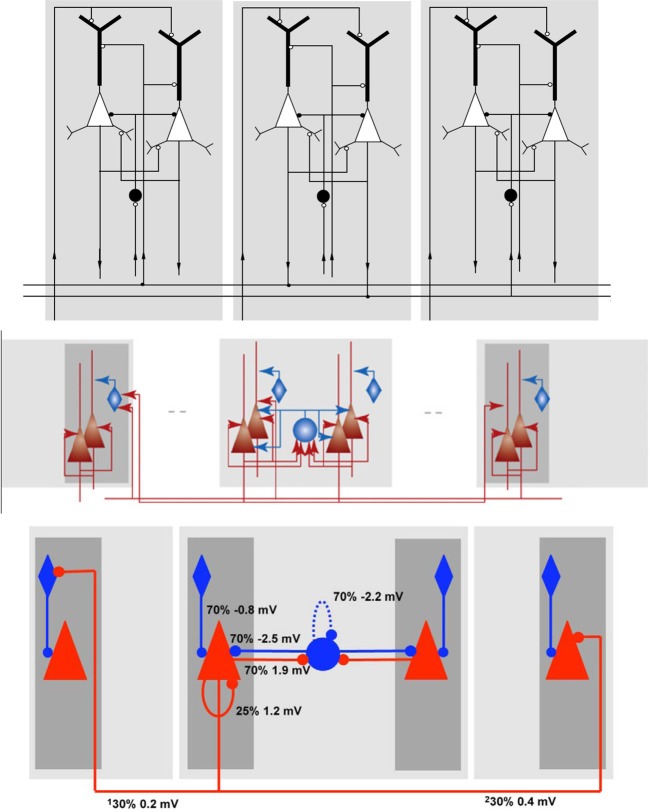
**Schematics of the Associative Memory Model in Layers II and III of the Neocortex, taken from Fransén and Lansner (1998), Lansner (2009), and Lundqvist et al. (2010) from top to bottom in this order**.

In Figure 6 three differently rendered schematics of the same NNS are shown to illustrate the diversity of visualizations. The underlying NNS is an *Attractor Network* model of cortical *Associative Memory* function based on *Cortical Columns* as described by Fransén and Lansner (1998) as a general model of the neocortical memory function. The model consists of populations in different layers, with excitatory as well as inhibitory projections in between the populations and organizational units. The schematics have been taken and are sorted in this order from Fransén and Lansner (1998) as Figure 6 top, from Lansner (2009) as Figure 6 center, and from Lundqvist et al. (2010) as Figure 6 bottom.

In the first schematic, Figure 6 top, the cortical layers are shown implicitly but were assigned names in the reference, with the higher cortical layers II/III at the top and the afferents from layer IV at the bottom. As the neurons are modeled as multi-compartment elements, the populations are rendered as complex shapes to illustrate the location of synaptic buttons. The neuron cell classes of the populations, although visually distinguishable in the image, are only specified in the reference. Projections between the populations show the direction via a circular pointer tip, where its filling denotes the connections excitation type: without filling for excitatory and with filling for inhibitory synapses. Solid highlighting demarcates a functional unit.

In Figure 6 center the ordering of the layers and the labeling of the areas remains the same as in the schematic before. As the referenced publication is mainly concerned with the principle of the model, the populations are now rendered in simpler shapes which are now shaded with a color gradient. The excitation type is indicated by colors with red for excitatory and blue for inhibitory. Arrows are again used for projections, but in contrast to the schematic of Figure 6 top the pointer tips now only indicate their direction.

The schematic in Figure 6 bottom is a slightly modified variant of the drawing before. The pointer tips are now rendered as circles, the gradient highlighting was replaced by plain highlighting and the projections are in addition visually attributed with the connection densities and average PSP amplitudes as measured at the soma.

### Comparison

By comparing the information found for the individual NNS descriptions, the common elements can be identified. A swift comparison already shows that the figures provide, although using different graphical styles, a common set of information. Table 1 lists the samples and summarizes what information were found for each in its figure as well as in its reference.

**Table 1:**
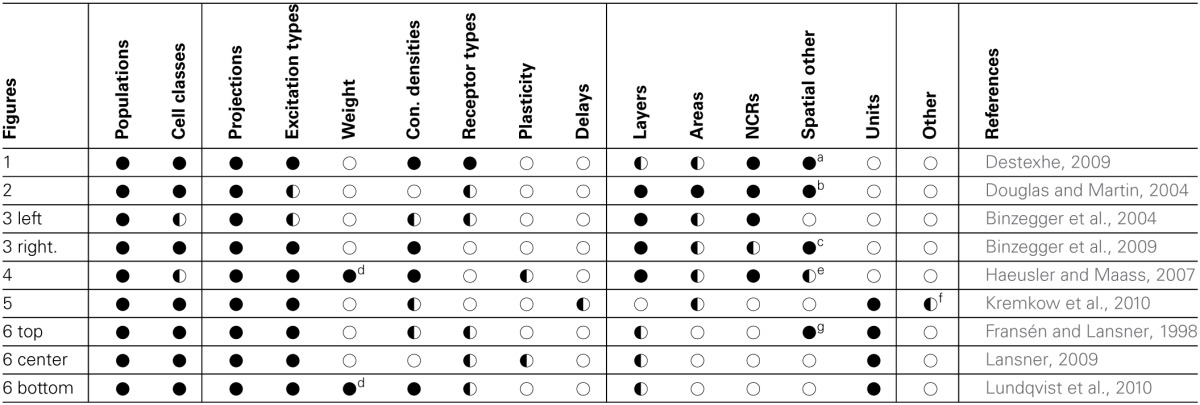
**Comparison of sample features; vertically grouped are neuronal, synaptic as well as spatial features of NNSs; horizontal lines in the data fields group examples that represent the same NNS concept; symbols denote that information is: ●—available in the original drawing and further explained in text,** 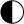
**—available in the referenced publication, ◌—not available**.

^a^Locality of synaptic connections.

^b^Locality of projection.

^c^Preferred layer of axonal innervation.

^d^Mean amplitude of PSP.

^e^Maximum intra-laminar distance of recording sites.

^f^Stimulus generating function.

^g^Location of synaptic buttons.

When looking at the table it can be seen that the authors of the figures used primarily populations and projections as the lowest level of abstraction. Single neurons were utilized only in Figure 1 and there presumably to illustrate the locality of the synaptic connectivity pattern as in the publication itself (Destexhe, 2009) the focus is foremost on the population's level. Therefore we will use populations and projections also as the lowest level of abstraction for *Neural Schematics*.

All the authors of the analyzed figures used cell classes either to label a population, transported this information via distinctive geometrical shapes, or provided this information in the corresponding reference. Consequently we will include the cell class information in the *Neural Schematic* concept. The same accounts for the synaptic excitation type which, is again mediated in different ways, but present.

As only Figure 1 provided the synaptic receptor types with the purpose of expressing the excitation type and other references provided that information only in text, we do not include these in *Neural Schematics*.

The strength of a projection can be indicated via the effective synaptic strength or the synaptic connection density. Only two of the figures included the average synaptic weights as the mean amplitude of the PSP, but all images or its references provided information on the synaptic connection density. To have at least one projection property expressing its strength visually we will include the synaptic connection density.

Dynamic attributes of synaptic connectivity such as plasticity or transmission delays were not present in any of the analyzed figures. However, Haeusler and Maass (2007) and Lansner (2009) provided information on plasticity, but only the first actually includes it in the model. Information on transmission delays in the model where solely given in Kremkow et al. (2010). We assume that the reason why the authors did not find it necessary to include these details in the figure is to put the focus of the graphical representation of the model on the static structure rather than its dynamic properties. We will therefore not include dynamic properties in the *Neural Schematic* concept.

Layer information is for all but one sample provided either as label of populations, via spatial positioning of populations, as backdrop to the schematic/graph, or given in the reference. Hence, this information will be included in the concept to be developed.

We will also incorporate areas and NCRs as this information is helpful in identifying the neuroanatomical correspondence, thus implicitly providing a spatial localization of a NNS. For the same reason units to logically group elements will also be included.

Some figures provided additional spatial information:

- the locality of the synaptic connectivity structure as present in Figure 1 and which will not be expressible in *Neural Schematics* as its level of abstraction is below the populations and projections,
- the locality of projections as shown in Figure 2 and which will be contained implicitly in the area and layer information,
- the preferred layer of axonal innervation of efferents leaving the NNS annotated via the population labels in Figure 3 right which will be rendered as projections into specific layers,
- the maximum intra-laminar distance of recording sites which is in that detail considered not relevant for a NNS model, and
- the location of synaptic buttons which is again below the level of abstraction of the *Neural Schematics*.

Only Figure 5 displayed uncategorized information that will not be present in a *Neural Schematic*.

### Defining symbols of neural schematics

For the set of elements that where chosen above as to be included in the *Neural Schematics* we will now define the graphical elements for a generic 2-D schematic representation of NNSs.

We begin with populations and projections as the basic elements to describe a NNS. Subsequently, elements for layers, areas, NCRs, and units are introduced to annotate further structural information to the *Neural Schematics*.

Following the definition guidelines given above, no features of graphical elements, i.e., the size of a shape or the thickness of a line, will be scaled according to attribute values to keep the *Neural Schematics* concept as simple as possible. However, lines might in general be of arbitrary thickness but proportional for all elements.

**Figure 7:**
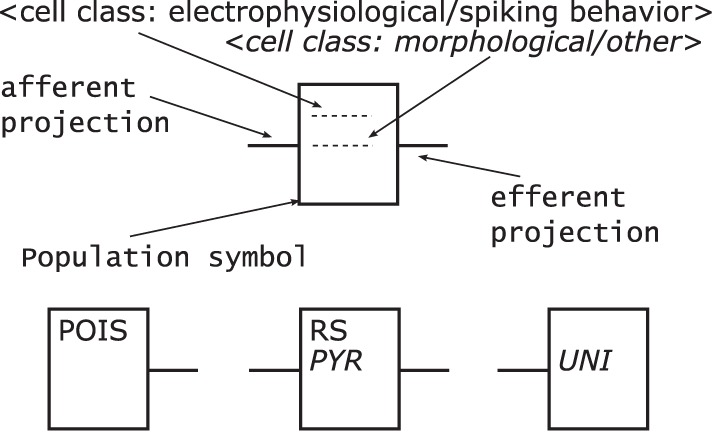
**A population object template shown at the top and three population examples at the bottom.** Following the template, a population is rendered as a rectangle and labeled with the electrophysiological cell class and/or the morphological type of its neurons; the afferent projections enter a population element on the left side relative to its label and the efferent projections leave it on the right side.

**Figure 8:**
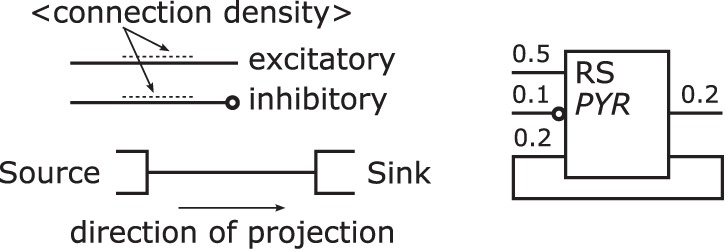
**Projection template shown on the left and examples on the right.** Following its template a projection is rendered as a line and might be labeled with its connection density, its direction is defined by the convention on which side projections enter/leave populations. An inhibitory projection is marked by an empty circle at its end.

**Figure 9:**
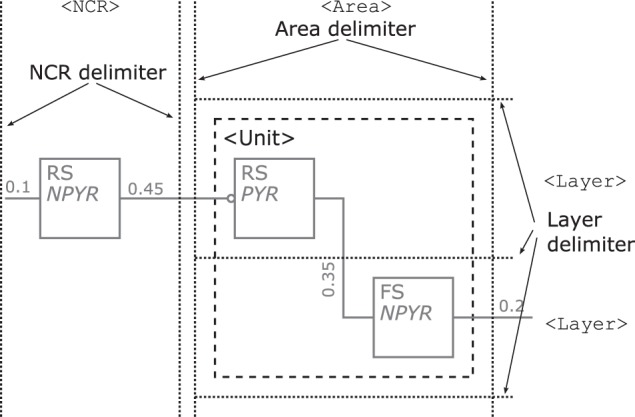
**Illustration of the rendering scheme for cortical layers, areas, NCRs, and functional units.** Layer boundaries are demarcated by dotted horizontal lines as shown on the right and any Area or NCR is differentiated by vertical lines. Layer, Areas, and NCRs share the same dotted line style. A dashed square, here grouping the two populations within the Area, represents a functional unit.

Populations—are represented by rectangles of arbitrary size and proportions, drawn with solid lines as shown in Figure 7 top. Although in the samples analyzed circles are the preferred shape for a population, we choose the rectangle using the graphical symbol for a logic function (IEEE, 1984) as a template as it enables the possibility to define a side for afferents and efferents.
Furthermore, as the number of categories for cell classes is currently not definite, see Markram et al. (2004) for example, we suggest a consistent naming scheme for populations rather than introducing distinct shapes as in Figure 5 or 6 for the variety of population types. A naming of populations based on the cell classes differentiated by the morphology and electrophysiological properties of the cells in a population combines the naming conventions used in some of the analyzed samples. A population is hence named after its neuron's electrophysiological class or a name describing its spiking behavior in normal typeset and/or its neurons morphological type as well as any other descriptive class in italic typeset. Other attributes of a population, as reasoned above, are not shown in its graphical representation.
Populations are the sources and/or sinks of projections which originate at the right hand side relative to the label of a population symbol and terminate at its left hand side. Figure 7 bottom provides three examples of populations as described above. On the left is a stimuli population named POIS which might generate Poisson distributed pulses, in the middle a population of cells with RS behavior and *Pyramidal* (PYR) morphology thus also named PYR, and on the right a population of motor cells with *Unipolar* (UNI) morphology thus named UNI.
Projections—connect populations as solid lines as shown in Figure 8 left. To denote the excitation type of a projection, an empty circle is added at the termination of an inhibitory afferent to a population similar to a negated input to a graphical symbol for a logic function (IEEE, 1984). Colors are not applied as to first keep the concept as simple as possible and, second, although common, their use is ambiguous, e.g., some use red for the excitatory type as in Figure 5 or 6 while others use the same color for the inhibitory type as in Figure 4. In addition and on the contrary to all the analyzed samples, no arrow ends or similar shapes are required to signal the direction of a projection as they are already directed by the defined sides for incoming and outgoing connections on a population.
As reasoned in the comparison, the connection density is used as a means of expressing a projection's strength as a label, if given. Other attributes of a projection, as mentioned above, are not shown in its graphical representation.
Unlike the connections in electrical circuitry projections, these connections are one-to-one, i.e., they do not branch or join. Projections that have no origin are considered inputs to the NNS and projections that do not terminate represent outputs of a NNS, respectively. A population might project onto itself the same way as it projects onto other populations.
Figure 8 right depicts an example of a population with its projections; it shows to the left hand side of the population symbol an excitatory inbound projection with a connection density of 0.5, an inhibitory incoming projection with a connection density of 0.1, and an incoming excitatory feedback loop that is the projection of a population onto itself with a connection density of 0.2. On the right hand side, in addition to the feedback loop, a second outgoing projection with a connection density of 0.2 is displayed.
Layers—might be integrated in a NNS's graphical representation, if this information is present as dotted horizontal lines as in Figure 9. The layers are named on the right hand side of the Neural Schematic with roman numerals in uppercase letters of a monospace fontset in normal typeset. The ordering of the layers is highest cortical layer on top.
Areas—might be added to a *Neural Schematic* as dotted vertical lines to delimit them as in Figure 9. Areas might be partitioned by layer boundaries and are named above using a monospace fontset in normal typeset.
NCRs—are rendered in the same visual style as areas but must not contain layer boundaries.
Units—provide the option to cluster populations functionally. A unit is represented by a rectangular shape of arbitrary size and proportions, rendered using a dashed line style. Projections to that logical unit may enter or leave the unit at arbitrary sides. A unit's name is determined by its function and set in normal typeset. A unit might not be a pure hierarchical element as it can house sub-units along with populations, as for example in Figure 6 bottom.

### Samples redrawn

Three of the samples are now redrawn with the information of the original figures combined where applicable with the details given in the references to illustrate the *Neural Schematics* concept of a unified formal 2-D graphical representation of NNSs.

**Figure 10:**
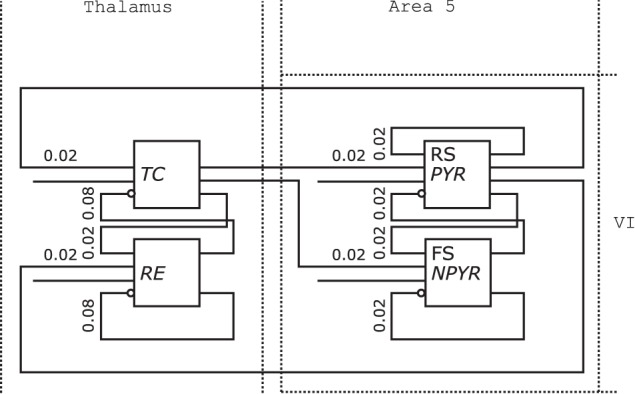
**Neural Schematic of Thalamocortical regions, following Destexhe (2009)**.

Figure 10 shows the redrawn NNS of Figure 1 from Destexhe (2009). The boxes of the original drawing dissolved into the background elements of the Thalamus as NCR without layers on the left and the Layer VI of Area 5 on the right.

The populations were renamed as listed in Table 2 according to the naming scheme established by the *Neural Schematics* concept. It is distinguished between the electrophysiological and morphological cell classes with the information given either by the original schematic or Destexhe (2009) to: RS/*PYR* for *PY* as the cells with a *Pyramidal* (PYR) morphology and RS behavior, FS/*NPYR* for *IN* as cells with a *Non-Pyramidal* (NPYR) morphology and FS behavior, while *TC* and *RE* are used as general descriptive classes.

**Table 2:** **Relabeling of the populations of Figure 1: left the original label, middle the electrophysiological or spiking class, right the morphological or other class**.

**Original**	**Elektrophys./spiking**	**Morph./other**
PYR	RS	PYR
IN	FS	NPYR
TC	–	TC
RE	–	RE

The connection densities added to the projections are now expressed in relative numbers. The synaptic receptor types of the projections are not incorporated in the redrawn figure since projections for *Neural Schematics* fall in either the excitatory or inhibitory category. Additional projections for the stimuli were included in the *Neural Schematic*.

**Figure 11:**
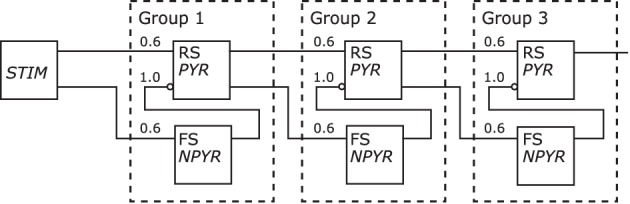
**The Neural Schematic of a Synfire Chain with Feed Forward Inhibition, after Kremkow et al. (2010)**.

Figure 11 depicts the redrawn model of Kremkow et al. (2010) with its original shown in Figure 5. The population labels were extended with the morphological cell class to RS/*PYR* and to FS/*NPYR*. The projections now show the connection density as given in the referenced publication. Details on the stimulus as shown in the original schematic are no longer as defined for the *Neural Schematics*. In contrast to the original figure, the NNS in the *Neural Schematic* is drawn with three groups, representing logical units instead of an unspecific number of these.

**Figure 12:**
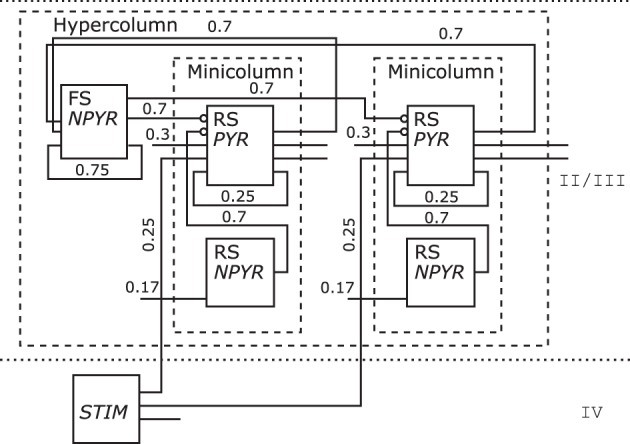
**The Neural Schematic of the Associative Memory model, following Lundqvist et al. (2010)**.

One *Hypercolumn* unit of the schematic in Figure 6 bottom of the Associative Memory model of the neocortical Layers II/III was redrawn as shown in Figure 12. As only layer information for that NNS is given, no area is shown. Populations are grouped to *Minicolumn* units that are housed along with a further population in a *Hypercolumn* unit.

The populations where renamed as listed in Table 3 to RS/*NPYR* for *RSNP* as the *Layer II/III Inhibitory Non-Pyramidal* or *Double-Bouquet* cells, FS/*NPYR* for *Layer II/III Inhibitory Non-Pyramidal* or *Basket* cells for *lateral inhibition*, to RS/*PYR* for the *Layer II/III Pyramidal* cells, and *Layer IV Input* abstracted as *STIM*. The information on synaptic connection density is shown as in the original figure; however, the synaptic strength represented by the mean PSP is no longer displayed.

**Table 3:** **Relabeling of the populations for Figure 12: left the original label, middle the electrophysiological or spiking class, right the morphological or other class**.

**Original**	**Elektrophys./spiking**	**Morph./other**
RSNP	RS	NPYR
Basket	FS	NPYR
PYR	RS	PYR
–	–	STIM

### Application

The *Neural Schematic's* level of abstraction with its focus on populations and projections rather than single neurons and synapses advocates its application to the visual design of NNSs, thus enabling CAD concepts within neuromorphic engineering. Modeling and simulation software applications such as neuroConstruct (Gleeson et al., 2007) or Thopographica (Bednar, 2009) already offer the possibility to design or edit neural network models via a graphical representation. The visualizations offered by these tools, however, are neither schematics nor as general as *Neural Schematics*.

**Figure 13:**
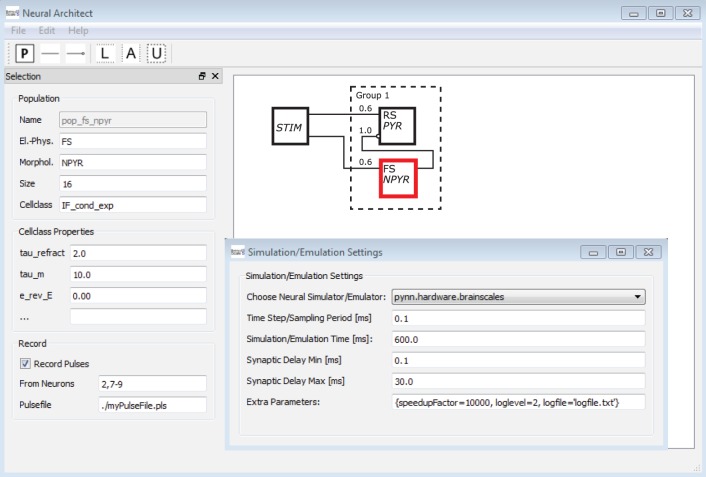
**A mock-up of a *Neural Schematics* application.** A software here named *Neural Architect* is intended to model NNSs and setup experiments for simulations on neuromorphic software simulators or emulations on neuromorphic emulator hardware, further description in text.

To underline the argument, a mock-up of an application^4^ is shown in Figure 13. A software tool here named *Neural Architect* might utilize *Neural Schematic* views to model NNSs and set up experiments for simulations/emulations. The GUI^5^ of the *Neural Architect* includes an editor to visually design a NNS, as can be seen on the right side in the background. Selecting a single *Neural Schematic* element, here a population object might allow the attribution of information to the element or modify its attributes via an entry mask as shown on the left side. Additional menus of the application might handle the experiment setup such as the simulator/emulator settings, the probing, or the data streaming of results.

Nordlie et al. (2009) assume that *“[o]ne reason [that textual descriptions of NNSs such as PyNN have not] yet caught on as a means of widespread model exchange may be [the lack of] human-comprehensible model descriptions [such as figures] that might be included in publications”* (cf. p. 16). The proximity of the PyNN language's sub-set of elements for the structural description of NNSs to the *Neural Schematics* elements qualifies it to complement *Neural Schematics* as its preferred textual representation to stream model information.

However, as the structural information describable with *Neural Schematics* is not congruent with the PyNN capabilities, the additional information such as the layers or the areas might have to be annotated to the corresponding PyNN elements.

## Discussion

*Neural Schematics*, as presented, standardize the way concepts of NNSs are visually transported. By analyzing a set of samples of current NNS depictions and references for what their authors intended to communicate and how they presented them, it is ensured that the *Neural Schematics* concept includes required and generally accepted means to express NNSs. It is still yet to be proven, however, that the concept holds when applying *Neural Schematics* to other NNSs, such as the examined ones, e.g., Johansson and Lansner (2007), Izhikevich and Edelman (2008), Deco et al. (2010), and Wagatsuma et al. (2011).

Due to the rule of projections entering a population on the left side relative to its label, and leaving on the right, respectively, the routing of the projections might cause a *Neural Schematic* to be in that regard visually more complex than the original as is the case when comparing Figure 6 with its *Neural Schematic* in Figure 12. A possible way to reduce some of this visual complexity, although none of the analyzed drawings utilized comparable elements, might be the introduction of hierarchical units to enable a higher level of describing NNSs. Hierarchical units are functional modules that would underly the same routing constraints as populations and hide their enclosed elements. Visual intricacies as introduced by coloring, highlighting, scaling, or complex shapes, however, are removed from the original figures.

Nevertheless, as the concept was developed with the constraints that simplicity should dominate over visual distinctiveness and that the *Neural Schematics* should be general to visually express as many NNS concepts as possible in the same style, it does not claim to be visually superior when compared to other diagram styles and it is as such not intended to completely replace more detailed illustrations of a specific NNS.

With its level of abstraction, i.e., having Populations and Projections at the lowest level, the *Neural Schematics* suffice for the communication of NNS concepts and descriptions not explicitly requiring more detail, for example, its neuron models or the connection structure inside a projection. This is in line with the arguments given as *good model description practice* from Nordlie et al. (2009) as it might ease the comprehension of a concept when not showing attributes of populations or projections in the schematic, but rather complement the figures with tables or other means more suitable, providing additional information such as CPTs.

The *Neural Schematics* concept furthermore provides the possibility to augment the logical setup of a NNS with spatial information by adding layers, areas, NCRs, or implicitly provide spatial information by grouping populations to units. Although none of the analyzed figure samples provided other explicit spatial information at the *Neural Schematic's* level of abstraction, a three dimensional spatial positioning of NNS elements is arguable, especially in the CAD context. However, from the authors' point of view such an extension of the *Neural Schematics* developed in this paper is not inevitable as it would lead to a concept resulting in visual representations of NNSs that are rather less suitable for publishable figures.

## Conclusion

We developed and presented *Neural Schematics* as a new unified schematic representation of NNSs to further remove the obstacles faced when communicating the ideas and concepts of NNS models. For common features of NNSs the *Neural Schematics* provide elements that were developed based on a comparative analysis of a set of samples from published graphical depictions of NNSs. The practicability of the concept was illustrated with a subset of the analyzed samples rendered as *Neural Schematics*.

Due to its routing conventions, a *Neural Schematic* might seem confusing when first exposed to the concept, but with growing acceptance and adaption the *Neural Schematics* might in general be easier to read than interpreting a new style with each new figure of a novel or even the same NNS. As an example supporting that hypothesis we refer to schematics describing electronic circuitry which underly similar conventions. These standards introduced in the 1970's and 1980's have been around for more than three decades now and are utilized to represent schematics of electrical circuitry with millions of elements.

The authors hope that *Neural Schematics* might ease the communication of novel NNS concepts and, supported by software tools enabling the CAD concept of *Visual Programming* of NNSs by providing functionality similar to the sketched *Neural Architect*, boost the modeling sciences, just as standards for schematics of electrical circuitry certainly did for VLSI^6^ design.

However, Sejnowski et al. (1988) characterized models of NNSs as *“provisional framework[s] for organizing possible ways of thinking about the nervous system”* (cf. p. 54) after which models and modeling concepts continually change and so must the concepts that are built around them. As such, we do agree that the concept of *Neural Schematics* might undergo iterations in evolving or refining it and therefore encourage a broader discussion concerning its fields of application and welcome suggestions on modifications to keep or even improve its general applicability.

### Conflict of interest statement

The authors declare that the research was conducted in the absence of any commercial or financial relationships that could be construed as a potential conflict of interest.

## Footnotes

^1^Institute of Electrical and Electronics Engineers.

^2^We consider the excitation type a property of the synaptic connection rather than of the presynaptic neuron.

^3^Computer Aided Design.

^4^Realization using PyQt by www.riverbankcomputing.co.uk and Qt from qt.digia.com

^5^Graphical User Interface.

^6^Very Large Scale Integration.
